# Efficient Degradation for Raffinose and Stachyose of a β-D-Fructofuranosidase and Its New Function to Improve Gel Properties of Coagulated Fermented-Soymilk

**DOI:** 10.3390/gels9040345

**Published:** 2023-04-18

**Authors:** Zhou Chen, Yimei Shen, Jiangqi Xu

**Affiliations:** 1 School of Food and Health, Beijing Technology and Business University, Beijing 100048, China; 2 School of Light Industry, Beijing Technology and Business University, Beijing 100048, China

**Keywords:** gel properties, β-D-fructofuranosidase, coagulated fermented-soymilk, *Leptothrix cholodnii*

## Abstract

A novel β-D-fructofuranosidase gene was identified via database mining from *Leptothrix cholodnii*. The gene was chemically synthesized and expressed in *Escherichia coli*, resulting in the production of a highly efficient enzyme known as LcFFase1s. The enzyme exhibited optimal activity at pH 6.5 and a temperature of 50 °C while maintaining stability at pH 5.5–8.0 and a temperature below 50 °C. Furthermore, LcFFase1s exhibited remarkable resistance to commercial proteases and various metal ions that could interfere with its activity. This study also revealed a new hydrolysis function of LcFFase1s, which could completely hydrolyze 2% raffinose and stachyose within 8 h and 24 h, respectively, effectively reducing the flatulence factor in legumes. This discovery expands the potential applications of LcFFase1s. Additionally, the incorporation of LcFFase1s significantly reduced the particle size of coagulated fermented-soymilk gel, resulting in a smoother texture while maintaining the gel hardness and viscosity formed during fermentation. This represents the first report of β-D-fructofuranosidase enhancing coagulated fermented-soymilk gel properties, highlighting promising possibilities for future applications of LcFFase1s. Overall, the exceptional enzymatic properties and unique functions of LcFFase1s render it a valuable tool for numerous applications.

## 1. Introduction

β-D-fructofuranosidase (EC 3.2.1.26) is a highly polymorphic protein that belongs to the glycoside hydrolase (GH) families 32. Its primary function is to catalyze the α1↔2β glycosidic linkage in molecules containing fructosyl moieties and is commonly known as invertase/sucrase, which inverts sucrose into an equimolar mixture of D-glucose and D-fructose [1]. However, β-D-fructofuranosidase also has the potential to cleave the fructosyl moieties from raffinose and stachyose, which are linked to melibiose/mannotriose and fructose with α1↔2β glycosidic linkage in molecules, and invert them into melibiose and mannotriose, respectively. Raffinose and stachyose are the primary concentration of galacto-oligosaccharides in soybean, which are the typical flatus-producing factors that lead to flatulence in the human gut [2]. The direct hydrolysis of raffinose/stachyose by β-D-fructofuranosidase eliminates these flatulence factors and produces several functional melibiose/mannotriose in soymilk [3]. Melibiose, a reducing disaccharide, has gained considerable attention since the early 21st century due to its beneficial attributes [3]. It promotes calcium absorption in the intestines, helps to cure atopic dermatitis [4,5], suppresses the Th2 response, inhibits aggregation-mediated neurodegenerative disorders as well as polyglutamine-mediated diseases [6,7], and can be used as a high-value additive in human functional foods and pharmaceuticals to maintain and promote good health [8]. These findings have gained much attention from scholars, but limited public literature exists on suitable β-D-fructofuranosidases with this special activity.

β-D-fructofuranosidases are widely used in the food industry and are present in a diverse range of organisms, including plants, animals, bacteria, yeasts, and filamentous fungi [9]. Microbial sources are highly desirable due to their advantages in industrial fermentation, such as high production rates, short fermentation periods, and good adaptability to varied environments. Yeasts are the primary producers of β-D-fructofuranosidase [10,11] followed by molds [12,13,14] and some bacteria [15,16]. However, most of these commonly used microbial β-D-fructofuranosidases are highly efficient in catalyzing sucrose with limited research conducted on their enzymatic activity towards raffinose or stachyose [3,17,18,19]. To date, only a few β-D-fructofuranosidases have been identified to possess this unique activity, including those from *Microbacterium trichothecenolyticum* [3], *Bacillus* sp. HJ14 [20], *Microbulbifer* rFF33, and *Microbulbifer* rIN33 [21]. Moreover, several levansucrases, which belong to another type of β-D-fructofuranosidase, have been found to be active toward raffinose [18]. Only one publication evaluated the practical potential of these specialized β-D-fructofuranosidases in real food applications [3]. The characterized InvDz13 was found to efficiently hydrolyze raffinose and produce melibiose during soymilk processing [3]. Therefore, additional resources on these distinct β-D-fructofuranosidases and their potential applications in diverse food industries are urgently required.

In this study, a new bacterial species, *Leptothrix cholodnii* SP-6, which has not been previously reported to have any glycoside hydrolase activity, was identified. Then, a unique β-D-fructofuranosidase gene from this strain was discovered, and its heterologous expression, purification, and catalytic properties were further characterized. Moreover, this enzyme’s ability to hydrolyze raffinose and stachyose as well as its potential application in soybean processing was finally evaluated.

## 2. Results and Discussion

### 2.1. Screening, Syntheting, and Cloning the β-D-Fructofuranosidase Gene from L. cholodnii

**Figure 1:**
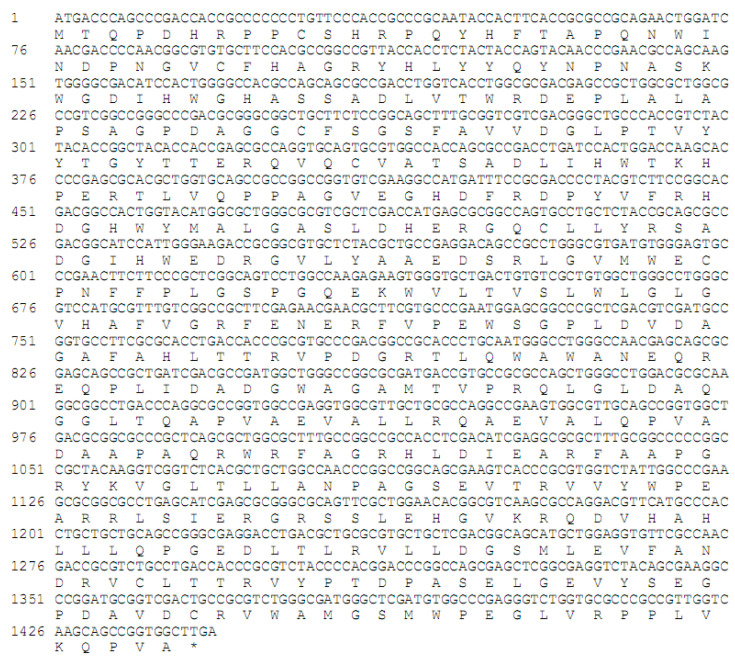
Nucleotide and deduced amino acid sequences of the full-length cDNAs and flanking regions of LcFFase1s.

With the rapid progress of bioinformatics, an increasing number of functional genes are publicly available, and the screening and rapid acquisition of these functional genes through gene repositories has become an efficient method for enzyme acquisition. In this study, we identified and obtained a newly registered β-D-fructofuranosidase gene (*LcFFase1s*) from the NCBI database, which originated from *L. cholodnii*. The amino acid and nucleotide sequences of the *LcFFase1s* gene are provided in Figure 1. The gene is 1443 bp and can encode 480 amino acids. The predicted molecular weight of the encoded protein is 53.3 kDa, and its predicted *p*I is 5.60. Using the protein sequences of other registered β-D-fructofuranosidases in the database, we performed a BLAST homology comparison to analyze LcFFase1s for homology with these proteins. The results showed that LcFFase1s had the highest similarity with the β-D-fructofuranosidase from *Deinococcus hopiensis* KR-140 (SMB85143.1) followed by the β-D-fructofuranosidases from *Phototrophicus methaneseepsis* (WP_195170317.1) and *Chloroflexus islandicus* (WP_082908989.1). However, the similarity between LcFFase1s and these enzymes was less than 70%, indicating that LcFFase1s has a high degree of novelty.

Microorganisms are commonly utilized in various industrial productions due to their unique biological advantages. The production of modern enzyme preparations, for example, primarily relies on microbial fermentation. As such, the exploration of microbial resources for enzyme production has always been a key focus of this industry. According to statistics, the industrial production of β-D-fructofuranosidase largely relies on *Aspergillus* spp. [22,23,24], *Bacillus* spp. [16,19], and *Bifidobacterium* spp. [25] along with a few other microorganisms, such as *Candida* spp. [26], *Cunninghamella echinulata* [27], and *Microbacterium trichothecenolyticum* [3]. Given the urgent demand for industrial production, the exploration of superior microbial resources for enzyme production remains a popular topic in enzyme research. Herein, we report the discovery of a β-D-fructofuranosidase gene from *Leptothrix cholodnii*, from which we successfully cloned the recombinant enzyme LcFFase1s. Our studies confirmed the enzyme’s excellent application potential for industrial applications. *L. cholodnii* is a newly discovered species, and currently, there are no reports on its secretion of glycoside hydrolases, particularly β-D-fructofuranosidase. Therefore, in-depth research on β-D-fructofuranosidase derived from *L. cholodnii* holds significant value for the development of new β-D-fructofuranosidase resources.

### 2.2. Purification, SDS-PAGE Analysis, and Enzyme Assay of LcFFase1s

**Figure 2:**
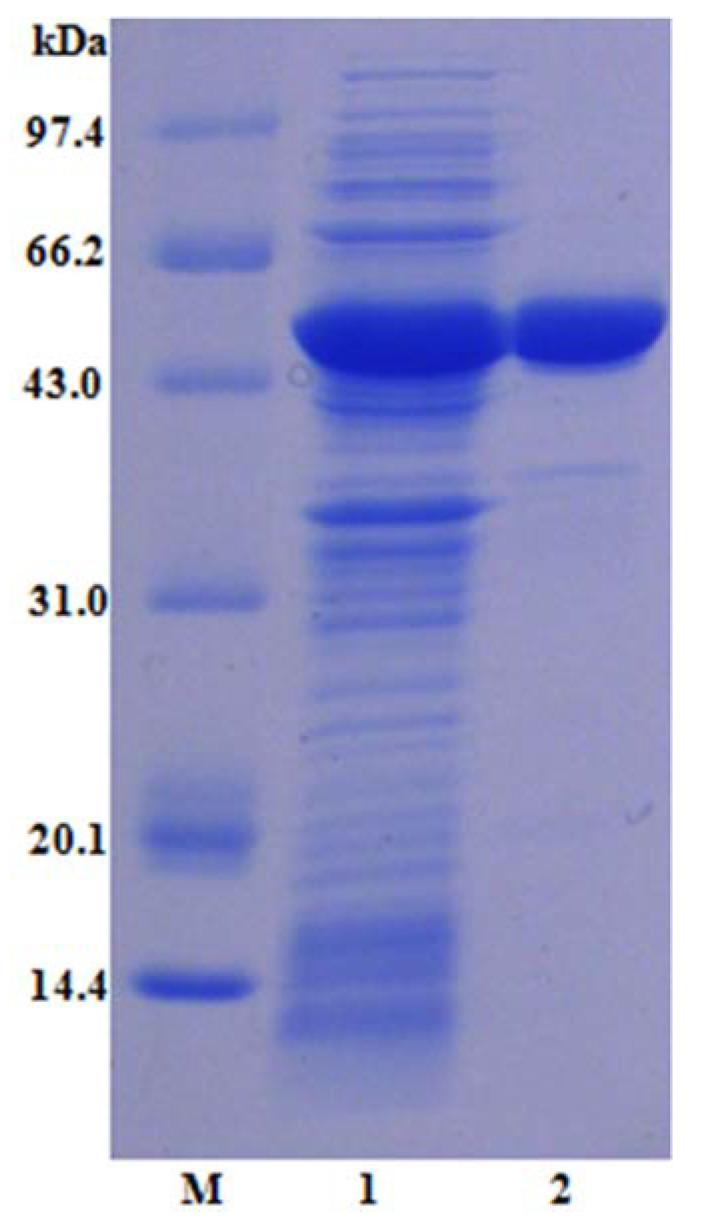
SDS-PAGE analysis of purified LcFFase1s. Lane M, low molecular weight standard protein markers; lane 1, crude lysate; lane 2, purified LcFFase1s.

The *LcFFase1s* gene was chemically synthesized and successfully transformed into an *E. coli* host, resulting in the expression of β-D-fructofuranosidase. Ni-IDA affinity chromatography was utilized as a purification method, resulting in the rapid obtainment of electrophoretic-grade pure enzyme LcFFase1s (Figure 2). A summary of the purification progress is presented in Table 1. The recombinant enzyme LcFFase1s displays a single band with an apparent molecular weight of approximately 53.4 kDa on SDS-PAGE. The specific activity of LcFFase1s increased from the initial value of 45.6 U/mg to 64.9 U/mg following the purification progress, resulting in a purification fold of 1.4-fold.

Upon analysis, it was determined that the LcFFase1s protein belongs to the class of typical bacterial β-D-fructofuranosidases. These enzymes typically exhibit molecular weights ranging from 50 kDa to 100 kDa, as exemplified by *Bifidobacteriaceae lactis* DSM10140T (59.4 kDa) [28], *Bacillus subtilis* LYN12 (66 kDa) [19,29], and *Microbacterium trichothecenolyticum* (approximately 66.2 kDa) [3]. In contrast, certain fungal β-D-fructofuranosidases display higher protein sizes, such as *Aspergillus niger* (116 kDa) [30] and *A. oryzae* S719 (95 kDa) [31].

### 2.3. Enzymatic Characterization of the Recombinant LcFFase1s

**Figure 3:**
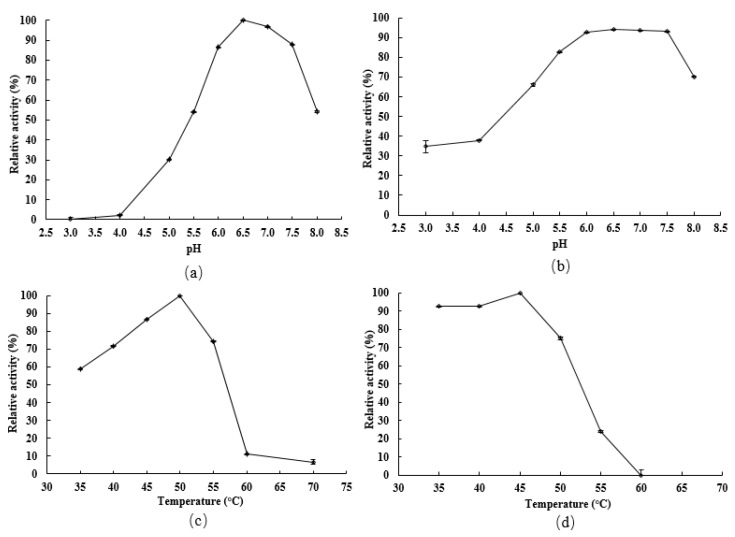
pH and temperature profiles of LcFFase1s. Effect of pH on the activity (**a**) and stability (**b**) was performed at 40 °C in 50 mM sodium hydrogen phosphate–citrate buffer. The remaining activities were measured after incubation for 30 min at 40 °C over various pH ranges. Effect of temperature on the activity (**c**) and thermostability (**d**) of LcFFase1s was determined at temperatures ranging from 25 °C to 70 °C in 50 mM sodium hydrogen phosphate–citrate buffer (pH 6.5).

**Figure 4:**
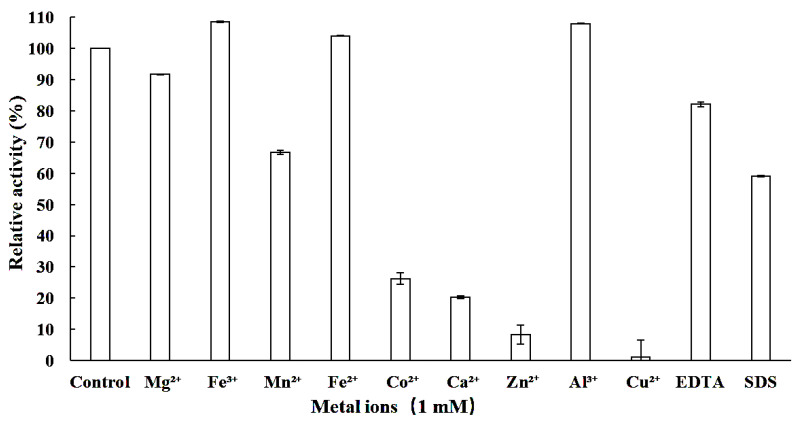
Effect of various metal ions on the β-D-fructofuranosidase of LcFFase1s.

This study initially investigated the impact of pH on both the catalytic activity and protein stability of the recombinant enzyme LcFFase1s. The results showed that the enzyme displayed maximum catalytic activity at pH 6.5 (Figure 3a) and retained more than 50% of its activity in the pH range of 5.5–8.0 (Figure 3b). Moreover, LcFFase1s exhibited remarkable stability at different pH levels with more than 70% residual activity after incubation for 30 min in the pH range of 5.5–8.0. Subsequently, the effect of temperature was studied, and it was found that LcFFase1s showed optimal catalytic activity at 50 °C (Figure 3c) while retaining more than 75% of its activity at temperatures below 50 °C (Figure 3d). Furthermore, we investigated the effects of different metal ions on the catalytic activity of LcFFase1s enzyme (Figure 4). We found that different ions had significant effects on the enzyme activity of the recombinant enzyme with Fe^3+^ and Al^3+^ promoting enzyme catalytic activity. In contrast, Mn^2+^, Co^2+^, Ca^2+^, Zn^2+^, and Cu^2+^ exhibited varying degrees of inhibition on the enzyme activity with inhibition rates of 33.4%, 73.8%, 79.8%, 92.6%, and 98.9%, respectively. Fe^2+^ and Mg^2+^ had almost no effect on enzyme activity. In addition, the effects of the metal chelator EDTA and surfactant SDS on enzyme activity were also evaluated. We found that EDTA only slightly interfered with enzyme activity, while SDS significantly inhibited enzyme activity, resulting in a residual enzyme activity of LcFFase1s at 59.0%.

The catalytic activity of β-D-fructofuranosidase is primarily determined by its catalytic region, which can be affected by various factors, such as pH, temperature, ions, and compounds. Thus, it is essential to comprehensively investigate the impact of these factors on enzyme catalysis. An optimal pH environment can significantly enhance enzyme activity, and LcFFase1s exhibited the highest enzyme activity at pH 6.5 with over 50% enzyme activity within a pH range of 5.5–8.0. Most bacteria-secreted β-D-fructofuranosidases have an optimal pH within a range of 6.0–8.0 [11,20,32]. In contrast, most fungal β-D-fructofuranosidases exhibit the lowest enzyme activity at lower pH values, such as *A. thermomutatus* [24], *A. terreus* [33], and *A. niveus* [34]. Moreover, acidic or alkaline environments can challenge enzyme protein structure stability, which limits their potential applications. However, LcFFase1s can maintain protein structure stability within a pH range of 5.5–8.0 with over 70% residual enzyme activity after storage within this range.

Temperature is a critical factor that can affect enzyme catalytic activity by inducing structural changes in their catalytic regions and active sites. LcFFase1s has been identified as a mesophilic enzyme with an optimal temperature of 50 °C, which is higher compared to β-D-fructofuranosidases from typical bacterial sources [28,35,36,37]. For instance, β-D-fructofuranosidases from *B. subtilis*, *Microbacterium trichothecenolyticum*, and *Synechocystis* spp. exhibit optimal temperatures of 40 °C, 35 °C, and 30 °C, respectively [3,29,32]. However, certain β-D-fructofuranosidases secreted by microorganisms, such as those from *A. thermomutatus* [24] and *A. terreus* [33], have optimal temperatures as high as 60 °C, indicating that they are thermophilic enzymes that require more heat to maintain their enzymatic activity. Enzymes are bioactive molecules, and their protein stability is generally temperature sensitive. LcFFase1s has been shown to maintain protein stability at temperatures of 50 °C and below, which is comparable to the thermal stability of some β-D-fructofuranosidases from bacteria and fungi, such as those from *Gongronella* sp. w5 [17], *B. longum* [25], *A. thermomutatus* [24], and *A. niveus* [34]. Good protein stability is a critical factor in ensuring normal catalytic activity of enzyme molecules.

Recent research has highlighted the critical role of metal cations in supporting enzyme catalysis, and various ions can interfere with enzyme activity in practical applications. Therefore, assessing the interaction between enzyme molecules and metal ions is of paramount importance [38]. For LcFFase1s, Mn^2+^, Co^2+^, Ca^2+^, Zn^2+^, and Cu^2+^ have been found to significantly inhibit its enzymatic activity, and these ions are also common inhibitors of β-D-fructofuranosidases from other sources, such as InvDz13 from *Microbacterium trichothecenolyticum* [3], GspInv from *Gongronella* sp. w5 [17], and β-D-fructofuranosidases from *B. breve* UCC2003 [36]. However, studies have also shown that Mn^2+^ and Ca^2+^ can enhance enzyme activity, as observed in InvDz13 [3], β-D-fructofuranosidases from *A. thermomutatus* (122.8%) [24], and *B. subtilis* (127.2%) [29]. Fe^3+^ has also been found to effectively enhance the catalytic activity of LcFFase1s, similar to the characteristics of β-D-fructofuranosidase from *A. sojae* [38]. EDTA, a common metal chelating agent, can be used to verify whether the enzyme’s catalytic activity depends on metal ions. The results of studies have indicated that EDTA has a minimal effect on the enzymatic activity of LcFFase1s, suggesting that the enzyme does not rely on any specific metal ion for catalysis [24]. In contrast, the surfactant SDS can significantly limit the activity of LcFFase1s, possibly by altering the enzyme’s structure or affecting its catalytic activity center at a certain concentration, as observed in β-D-fructofuranosidase from *A. sojae* [39].

### 2.4. Resistance to Proteolytic Degradation of LcFFase1s

**Figure 5:**
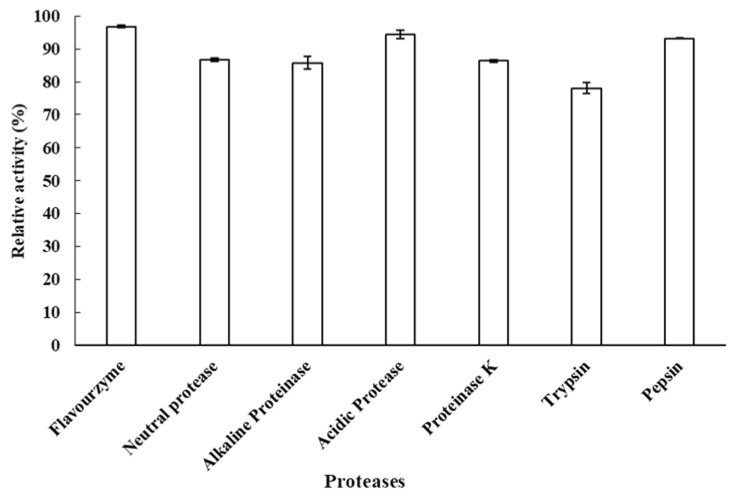
The ability of β-D-fructofuranosidase LcFFase1s to resist various proteases.

This study sought to assess the protease resistance of the recombinant enzyme LcFFase1s against commercially available proteases. The findings revealed that, when administered at a dose of 10 U/mL, the chosen seven proteases were unable to hydrolyze the LcFFase1s protein within a period of 30 min. Moreover, the residual enzyme activity of the protein remained at or above 87.0% (Figure 5).

LcFFase1s is a protein that is theoretically vulnerable to hydrolysis by specific proteases, resulting in the breakdown of the protein into smaller peptide molecules, which would significantly impair its β-D-fructofuranosidase catalytic activity. In the food processing industry, enzyme preparations are commonly used in the form of multi-enzyme complexes, which include proteases. However, this presents a significant challenge for other enzymes, underscoring the importance of identifying enzymes that possess strong resistance to proteolytic degradation for practical production purposes. Fortunately, our study identified LcFFase1s and demonstrated its potential for application by revealing its ability to tolerate hydrolysis by seven commonly used commercial proteases. This resistance to proteolysis can be attributed to the unique protein structure of the enzyme, which likely lacks the cleavage sites for these proteases in the key catalytic region. Similar phenomena have been observed in other glycoside enzymes, such as xylanases and glucosidase [38,40,41].

### 2.5. Ability of LcFFase1s to Hydrolyze Raffinose and Stachyose into Melibiose and Mantrionose

**Figure 6:**
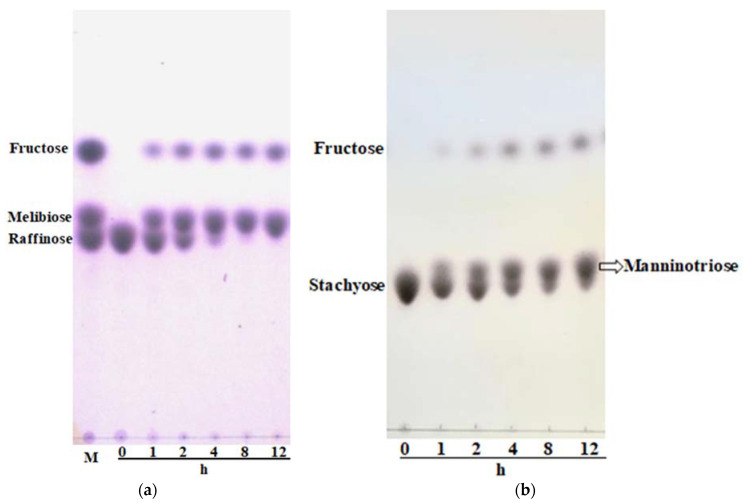
Hydrolysis of raffinose (**a**) and stachyose (**b**) by LcFFase1s. The reaction mixture containing 20 mg/mL of substrates in 50 mM sodium hydrogen phosphate–citrate buffer (pH 6.5) and LcFFase1s (5 U/mL) was incubated at 40 °C.

LcFFase1s is a β-D-fructofuranosidase that displays high catalytic efficiency, enabling it to efficiently hydrolyze sucrose. Furthermore, it has the capacity to hydrolyze raffinose and stachyose, as illustrated in Figure 6. At a concentration of 5 U/mL, LcFFase1s entirely hydrolyzed raffinose and stachyose into fructose and melibiose or raffinose within a time frame of 8 h and 24 h, respectively.

β-D-fructofuranosidase exhibits a high level of substrate selectivity and can specifically hydrolyze α1↔2β glycosidic linkages. Sucrose, for instance, can be hydrolyzed to yield one molecule of glucose and one molecule of fructose. In nature, some oligosaccharides and polysaccharides contain sucrose molecule structures, such as raffinose and stachyose. Raffinose (Gal-α(1→6)-Glc-(α1↔2β)-Fru) consists of one molecule of galactose and one molecule of sucrose, while stachyose (Gal-α(1→6)-Gal-α(1→6)-Glc-(α1↔2β)-Fru) consists of two molecules of galactose and one molecule of sucrose. Therefore, theoretically, β-D-fructofuranosidase can hydrolyze the α1↔2β glycosidic linkages in raffinose and stachyose, releasing fructose. Through the study of the hydrolytic characteristics of LcFFase1s, we confirmed that β-D-fructofuranosidase has the ability to degrade raffinose and stachyose, and complete hydrolysis can be achieved. However, there are limited reports on the hydrolysis of raffinose and stachyose by β-D-fructofuranosidase in existing studies. Currently, only a few studies have mentioned the hydrolysis of raffinose by β-D-fructofuranosidase, such as enzymes derived from *M. trichothecenolyticum* [3], *Gongronella* sp. w5 [17], and *Leuconostoc mesenteroides* [18]. Reports on the hydrolysis of stachyose by β-D-fructofuranosidase are even rarer [19]. Therefore, the discovery of the unique hydrolytic characteristics of LcFFase1s provides a solid foundation for exploring the broader applications of this enzyme in the future. For example, LcFFase1s is expected to be used to eliminate flatulence factors, such as raffinose and stachyose, in legume-processed foods [41].

### 2.6. Effect on Gel Properties of LcFFase1s during the Process of Coagulated Fermented-Soymilk

**Figure 7:**
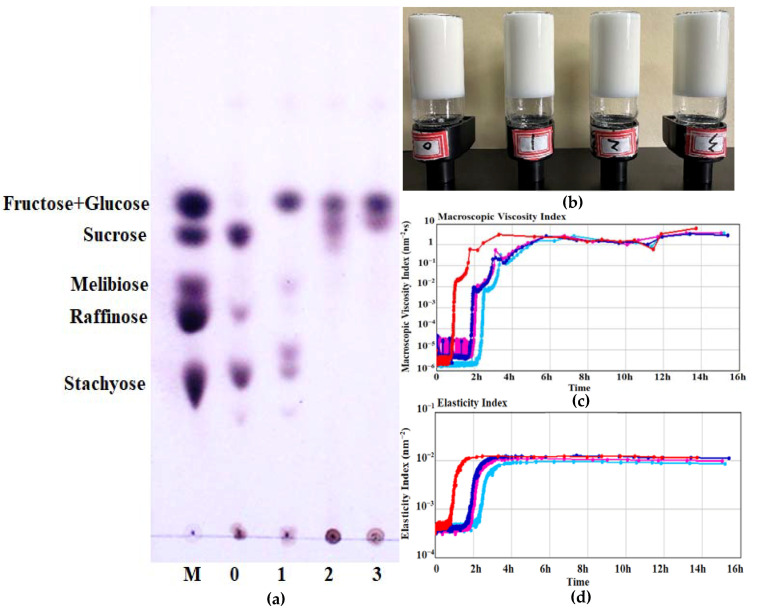
Ability of LcFFase1s to catalyze the hydrolysis of soybean (**a**) and effect of LcFFase1s on the gel properties (**b**–**d**) of coagulated fermented-soymilk.

Expanding upon the aforementioned findings, this study further explored the impact of LcFFase1s on the hydrolysis of stachyose and raffinose in defatted soybean as well as its role in the fermentation of coagulated fermented-soymilk. Results indicated that 5 U/mL of LcFFase1s effectively catalyzed the hydrolysis of stachyose and raffinose in defatted soybean, yielding fructose and melibiose or raffinose and achieving complete hydrolysis of stachyose (Figure 7a). Investigation of LcFFase1s’s effect on coagulated fermented-soymilk gel formation demonstrated that its addition did not alter the appearance, hardness, or viscosity of the soymilk gel (Figure 7b–d). Furthermore, LcFFase1s augmented the gel’s particle size with the size gradually decreasing as the enzyme dosage increased (Table 2).

Raffinose and stachyose are the oligosaccharides commonly found in legumes, and their high content can cause flatulence and digestive discomfort when consumed excessively, thereby affecting the quality of legume-based foods [3]. LcFFase1s efficiently hydrolyzes stachyose and raffinose in defatted soybean, which is beneficial in eliminating the flatulence factors in legumes and has potential applications in the processing of legume-based foods. Moreover, the degradation products, such as melibiose, are functional sugars that have potential health benefits, including the inhibition of aggregation-mediated neurodegenerative disorders and diseases mediated by polyglutamine [7,42]. These end-products have high value as additives in human functional foods and pharmaceuticals that promote and maintain good health [8].

Coagulated fermented-soymilk is a type of fermented dairy product that is made from a combination of soy milk and milk, which is fermented using lactic acid bacteria. This product offers a dual nutritional benefit from both soymilk and dairy milk. Our study shows that LcFFase1s not only eliminates common flatulence factors present in bean-based foods but also enhances the gelling properties of coagulated fermented-soymilk. Specifically, compared to the fermentation process of coagulated fermented-soymilk without enzyme addition, the addition of LcFFase1s helps maintain the hardness and viscosity of the fermented-soymilk during the gel formation process, which results in an aesthetically pleasing appearance and commercial quality of the fermented-soymilk product. On the other hand, our findings demonstrate that an increasing amount of LcFFase1s leads to a significant reduction in the gel particle size during the fermentation process of soymilk, resulting in finer gel particles that enhance the texture and smoothness of the fermented-soymilk. This change may be attributed to the alteration of sugar types in soymilk during fermentation induced by LcFFase1s addition, which subsequently affects the formation of the fermented-soymilk gel network structure [43]. Several other studies have corroborated the advantageous impact of diverse saccharides, including oligosaccharides, on protein gelation in legume-derived comestibles [44]. Moreover, investigations have demonstrated the capacity of these oligosaccharides to ameliorate protein gelling to a certain extent in various food matrices, such as casein in yogurt [45], egg white protein [46], and myofibrillar proteins in meat [47], while also altering the texture and rheological properties of the gel and potentially impacting the formation of its dense network structure.

## 3. Conclusions

The present study focused on the discovery and expression of a novel β-D-fructofuranosidase gene from *L. cholodnii*, which resulted in the production of the highly efficient enzyme LcFFase1s. This enzyme showed exceptional catalytic properties and demonstrated its potential applications in legume processing by efficiently hydrolyzing raffinose and stachyose. Furthermore, the addition of LcFFase1s during the fermentation process of coagulated fermented-soymilk resulted in a reduction in particle size, leading to a smoother texture without compromising the gel hardness and viscosity. This study is the first to report on the positive impact of β-D-fructofuranosidase on coagulated fermented-soymilk gel properties, opening up promising avenues for future applications of LcFFase1s.

## 4. Materials and Methods

### 4.1. Reagents

The glucose-oxidase kit utilized for activity assessment was obtained from Applygen Technologies Inc. (Beijing, China). Melibiose, raffinose, and stachyose were procured from Sigma (St. Louis, MO, USA) for thin-layer chromatography (TLC) analysis. Soybean meal utilized in this study was acquired from a local market in Beijing, China.

### 4.2. Gene Excavation, Cloning, and Expression of the β-D-Fructofuranosidase

A novel gene, named *LcFFase1s* and registered as β-D-fructofuranosidase (GenBank No: ACB35555.1), was identified and retrieved from the NCBI database. The codon usage of this gene was optimized, and the gene was chemically synthesized by Synbio Technologies in China. The resulting gene fragment was amplified with PCR using two primers, LcFFase1s-up and LcFFase1s-down, which contained *NdeI* and *XhoI* sites. The PCR products were purified, digested with *NdeI* and *XhoI*, and sub-cloned into the pET-28a (+) vector. The constructed plasmids were then transformed into *E. coli* BL21 (DE3) cells to successfully express the target gene. A native colony containing LcFFase1s of *E. coli* was selected and inoculated into LB medium supplemented with kanamycin (50 μg/mL) and incubated at 37 °C until the optical density reached 0.6–0.8. Expression of the recombinant protein was induced with 1 mM isopropyl β-D-thiogalactoside (IPTG), and the strains were cultured at 20 °C to accumulate crude β-D-fructofuranosidase.

### 4.3. Purification, Protein, and Catalytic Properties of Recombinant LcFFase1s

The harvested cells were suspended in lysis buffer A (50 mM Tris-HCl, pH 7.4) and sonicated to break the cell wall. The resulting supernatant containing the crude enzyme was collected for further purification after centrifugation. Affinity chromatography using a Ni-IDA column (0.8 × 10 cm) with gradient elution was employed for purifying the crude enzyme. The enzyme was loaded onto the column that was pre-equilibrated with buffer B (buffer A containing 20 mM imidazole), washed with buffer C (buffer A containing 50 mM imidazole), and eluted with buffer D (buffer A containing 200 mM imidazole) at a flow rate of 1.0 mL/min. The eluted fraction was collected and assessed for β-D-fructofuranosidase activity. The purification summary was calculated based on enzymatic activity and protein concentration, while protein bands during purification were monitored with 12.5% SDS-PAGE after staining with Coomassie brilliant blue R-250. The molecular weight of the purified protein was determined using low molecular mass standards.

### 4.4. Enzyme Assay and Protein Determination

The activity of β-D-fructofuranosidase was evaluated in 50 mM sodium hydrogen phosphate–citrate buffer (pH 6.5) at 40 °C. To elaborate, 20 µL of LcFFase1s was combined with 180 µL of sucrose (200 mg/mL) and incubated for 10 min. The glucose released was measured using the glucose-oxidase kit, and 1 unit (U) of enzyme activity was defined as the amount of the enzyme that liberated 1 μmol of glucose per minute. The protein concentrations were measured using the Lowry method with bovine serum albumin as a standard [48].

### 4.5. Characterization of the Recombinant β-D-Fructofuranosidase

The pH and temperature characteristics of LcFFase1s, including optimal pH and temperature as well as pH and thermal stability, were investigated. Specifically, the β-D-fructofuranosidase activity was measured in phosphate–citrate buffer at pH values ranging from 3.0 to 8.0, and the optimal pH was determined as the pH at which the enzyme displayed the highest activity. Similarly, the enzyme activity was assessed at various temperatures between 35 and 70 °C, and the optimal temperature was identified as the temperature at which the enzyme displayed the highest activity.

To evaluate the pH and temperature stability of LcFFase1s, the enzyme solution was incubated in phosphate–citrate buffer with different pH values (ranging from 3.0 to 8.0) or at various temperatures (between 35 and 70 °C) for 30 min. Subsequently, the solution was rapidly cooled in an ice bath, and the residual activity was determined using the standard assay.

### 4.6. Effect on β-D-Fructofuranosidase Activity of Cations In Vivo

The effect of various cations present in different application systems on the activity of LcFFase1s was examined. The enzyme was incubated with 1 mM cations at 40 °C for 30 min, and the remaining activity was determined using the standard assay.

### 4.7. Resistant Ability of β-D-Fructofuranosidase to the Hydrolysis of Proteases

The susceptibility of LcFFase1s to degradation by commercial proteases, such as Flavourzyme, Neutral protease, Alkaline proteinase, Acidic protease, Proteinase K, Trypsin, and Pepsin, was examined as a soluble protein. The enzyme was exposed to 1 mg/mL protease at 37 °C for 30 min followed by a determination of the residual activity of both the treated and untreated β-D-fructofuranosidase using the standard assay.

### 4.8. Hydrolysis Ability on Raffinose and Stachyose of LcFFase1s

Prepare 2% solutions of cottonseed gum and guar gum in phosphate–citrate buffer (50 mM, pH 6.5), respectively. Add β-D-fructofuranosidase to each solution to a final concentration of 5 U/mL and carry out enzyme-catalyzed reactions at 40 °C for different hydrolysis times (1 h, 2 h, 4 h, 8 h, 12 h, 24 h). Take samples at each time point and quench the enzyme activity by boiling the samples for 5 min. Analyze and detect the hydrolysis products using TLC under the following conditions: Spot the samples onto silica gel plates and develop them twice in a solvent system consisting of n-butanol: acetic acid: water (2:1:1 *v*/*v*/*v*); then, visualize the saccharides by heating the plates in an oven after spraying them with a mixture of methanol and sulfuric acid (95:5, *v*/*v*).

### 4.9. Effect on Gel Properties of LcFFase1s during the Process of Coagulated Fermented-Soymilk

The impact of β-fructofuranosidase LcFFase1s on the formation and gel properties of coagulated fermented-soymilk was investigated. A mixture of 20% soymilk and 80% milk was inoculated with 3% starter (Angel Yeast Co., Ltd., Yichang, China) and various concentrations of LcFFase1s and then fermented at 40 °C to produce coagulated fermented-soymilk. After fermentation, the gel properties of the coagulated fermented-soymilk, including the elasticity index and macroscosity index, were analyzed using an optical rheometer (RHEOLASER MASTER, LAB 6 MASTER).

### 4.10. Statistical Analysis

Data were expressed as the mean ± SD and subjected to one-way analysis of variance (ANOVA, SPSS version 10.0) to determine significant differences. These differences (*p* < 0.05) were reanalyzed with the least significant difference multiple-range test.

## Figures and Tables

**Table 1:** Summary of the recombinant β-D-fructofuranosidase (LcFFase1S) expressed in *E. coli*.

Purification Step	Total Activity	Protein	Specific Activity	Purification	Recovery
	(U) ^a^	(mg) ^b^	(U/mg)	Factor (-Fold)	(%)
crude supernatant	7724.2	169.6	45.5	1.0	100.0%
Ni-IDA	1289.1	19.9	64.8	1.4	16.7%

^a^ Activity was measured in 50 mM sodium hydrogen phosphate–citrate buffer (pH 6.5) at 50 °C using 20% sucrose. ^b^ The protein was measured with the Lowry method using BSA as the standard.

**Table 2:** Effect of LcFFase1s on the particle size of coagulated fermented-soymilk.

Concentration (U/mL)	0	5	10	20
Particle size (nm)	3814 ± 22.62	3486.5 ± 20.50	3188.5 ± 14.84	2347.5 ± 17.67

## Data Availability

Not applicable.
